# Development and Testing of the MyHealthyPregnancy App: A Behavioral Decision Research-Based Tool for Assessing and Communicating Pregnancy Risk

**DOI:** 10.2196/mhealth.7036

**Published:** 2017-04-10

**Authors:** Tamar Krishnamurti, Alexander L Davis, Gabrielle Wong-Parodi, Baruch Fischhoff, Yoel Sadovsky, Hyagriv N Simhan

**Affiliations:** ^1^ Department of Engineering and Public Policy Carnegie Mellon University Pittsburgh, PA United States; ^2^ Magee-Womens Research Institute Department of OBGYN and Reproductive Sciences University of Pittsburgh Pittsburgh, PA United States; ^3^ Department of Microbiology and Molecular Genetics University of Pittsburgh Pittsburgh, PA United States

**Keywords:** mhealth, pregnancy, premature birth, decision making

## Abstract

**Background:**

Despite significant advances in medical interventions and health care delivery, preterm births in the United States are on the rise. Existing research has identified important, seemingly simple precautions that could significantly reduce preterm birth risk. However, it has proven difficult to communicate even these simple recommendations to women in need of them. Our objective was to draw on methods from behavioral decision research to develop a personalized smartphone app-based medical communication tool to assess and communicate pregnancy risks related to preterm birth.

**Objective:**

A longitudinal, prospective pilot study was designed to develop an engaging, usable smartphone app that communicates personalized pregnancy risk and gathers risk data, with the goal of decreasing preterm birth rates in a typically hard-to-engage patient population.

**Methods:**

We used semistructured interviews and user testing to develop a smartphone app based on an approach founded in behavioral decision research. For usability evaluation, 16 participants were recruited from the outpatient clinic at a major academic hospital specializing in high-risk pregnancies and provided a smartphone with the preloaded app and a digital weight scale. Through the app, participants were queried daily to assess behavioral risks, mood, and symptomology associated with preterm birth risk. Participants also completed monthly phone interviews to report technical problems and their views on the app’s usefulness.

**Results:**

App use was higher among participants at higher risk, as reflected in reporting poorer daily moods (Odds ratio, OR 1.20, 95% CI 0.99-1.47, *P*=.08), being more likely to smoke (OR 4.00, 95% CI 0.93-16.9, *P*=.06), being earlier in their pregnancy (OR 1.07, 95% CI 1.02-1.12, *P*=.005), and having a lower body mass index (OR 1.07, 95% CI 1.00-1.15, *P*=.05). Participant-reported intention to breastfeed increased from baseline to the end of the trial, t15=−2.76, *P*=.01. Participants’ attendance at prenatal appointments was 84% compared with the clinic norm of 50%, indicating a conservatively estimated cost savings of ~US $450/patient over 3 months.

**Conclusions:**

Our app is an engaging method for assessing and communicating risk during pregnancy in a typically hard-to-reach population, providing accessible and personalized distant obstetrical care, designed to target preterm birth risk, specifically.

## Introduction

Preterm birth rates in the United States are on the rise, with approximately 1 of every 10 births occurring prior to 37 weeks of gestation [1]. These rates are also disproportionately high among some sociodemographic groups, reaching 1 in 6 among African-Americans [2], with greater prevalence among families living in poverty, regardless of race [3]. These patient groups are also often the hardest to reach due to limited access to and attendance at routine prenatal care.

The consequences of preterm birth are severe, and its causes are complex. Medical interventions for reducing preterm birth typically address one or a few risk factors in isolation, such as antibiotics for asymptomatic bacteriuria [4], 17-alpha hydroxyprogesterone caproate for a history of previous preterm birth, or cerclage for sonographically short cervix [5,6]. These interventions are also often predicated on medical testing, such as cervical measurement, blood-glucose testing, and serial blood pressure readings, which require both patient engagement and follow up. There are other seemingly simple precautions with potentially significant impact, such as the daily intake of a multivitamin during early pregnancy. However, it has proven difficult for health care providers to communicate even these recommendations effectively enough to secure sustained behavior change [7]. In this study, we demonstrate a behavioral intervention that engages pregnant women, providing them with information about risks related to preterm birth that can be identified without medical testing, as well as a suite of protective actions that they can utilize, with support from their health care providers.

In its Committee Opinion on Effective Patient-Physician Communication, the American College of Obstetricians and Gynecologists emphasizes the critical, important nature of effective and compassionate patient-provider communication, specifically noting the opportunities to provide such support with emerging information technologies [8]. Our intervention follows this strategy, as part of the move to provide patient services via mobile phone [9,10]. This strategy is possible because, even in the lowest income bracket, 86% of American adults own a mobile phone and three-fourths of this population own a smartphone, with similar ownership rates across racial and ethnic groups [11]. Moreover, as of 2015, almost 20% of all smartphone users had downloaded at least one pregnancy app [11]. Thus, in principle, apps offer a unique channel for communicating with patients, both for gathering information from them and for addressing their needs—if they can be engaged with the device and use its contents. Our intervention designs such an app, MyHealthyPregnancy (MHP) *,* using theory, results, and methods from behavioral decision research to provide personalized risk communication, specifically aimed at pregnant women at risk of not receiving such information due to routinely missing prenatal care. It is developed in collaboration with women from that population and then tested for feasibility in a 3-month trial. Although the ultimate implementation goal is to decrease preterm birth rates when used over the full duration of a pregnancy, here we report on the development process and the ability of the app to engage a hard-to-reach patient population. The results reported here address our preliminary research aims of (1) creating an app to engage an at-risk patient population, (2) increasing their attendance at prenatal care appointments, (3) identifying and communicating risk factors associated with preterm birth outcomes, and (4) encouraging risk-reduction behaviors and health promoting intentions.

## Methods

### App Development

To create an app to engage our target population, we followed the behavioral decision research paradigm [12-14] consisting of (1) normative research, (2) descriptive research, (3) prescriptive interventions, and (4) evaluation research. First, normative research identifies the need to make informed decisions based on the best available medical knowledge. Second, descriptive research characterizes women’s current beliefs, values, and constraints; Third, prescriptive interventions provide better information and reduce barriers to desired actions. Finally, evaluation research assesses the effectiveness of those interventions and the validity of the research upon which they rely, in pretests and a field test. User-centered design, informed by feedback from members of the target audience, was central to the process [15,16].

### Semistructured Interviews

Our normative research began by consulting with 4 medical expert informants in the field of maternal-fetal medicine and community informants from a diverse set of group (e.g., churches, non-profit organizations, women’s shelters, doula groups), before proceeding with our descriptive work. Descriptive work included in-depth, semistructured interviews with 5 women recruited through a church and a family support center in a neighborhood that met demographic criteria of high-risk pregnancy populations (ie, lower average household income, higher proportion of African-American residents, higher proportion of single marital status mothers).

Experts were questioned about the best ways to assess those preterm birth risks falling within their field of specialty (eg, our intimate partner violence experts were asked about which measures to use to assess IPV, as well as how frequently to measure IPV risk in the target group). Our pregnant participants were asked questions related to the causes of preterm birth risk, structured around the risks identified in the normative phase, as well as about barriers to prenatal care access (eg, “What are some things moms can do to avoid a preterm birth?” and “Has there ever been a time when you couldn’t make it to one of your appointments?”)

All semistructured interview participants were screened for eligibility (at least 18 years of age, neighborhood resident, currently pregnant, or with a child under the age of 1 year). Participants were interviewed between March and April, 2015. Although the sample size was smaller than typical for descriptive work using this approach [17,18], the issues faced by these women were sufficiently similar that few new ones emerged in succeeding interviews, leading us to move on to secure direct feedback from potential users of the app prototype [19].

We interpreted the interviews based on established medical science and clinical practice (normative analysis), as well as research into decision-making processes (descriptive research). Tables 1 and 2 outline critical preterm birth factors that we identified from the normative and descriptive analyses and the features we incorporated into the app to address each of them. These risk factors were pregnancy history (eg, prior preterm births, neonatal intensive care unit stays for previous births), weight gain trajectory (using the 2009 Institute of Medicine guidelines), smoking, alcohol consumption, illegal drug use, symptoms of preterm labor (eg, vaginal bleeding and fluid loss), intimate partner violence (assessed with the HITS Screening Tool that measures “hurt, insult, threaten, and scream”) [20], and depression (assessed with the Edinburgh Postnatal Depression Scale) [21]. This final app content was reviewed by the physician experts in our team for accuracy and potential clinical benefit. Our descriptive interviews revealed that transportation to appointments was a barrier to prenatal care, particularly among low-income patients. To lower this barrier, we incorporated free transportation using Uber into the app’s functionality.

**Table 1:** Behavioral risk factors identified in normative and descriptive research and addressed in the MyHealthyPregnancy app.

Pregnancy risk factor	Common challenge or misperception	Exemplar quote	App-based solution
Nutrition and weight gain	Avoidance of weight measurement Beliefs about nutrition	“I’m really into fitness and workout every day, so it’s depressing to me to see how much weight I’ve gained. So, I actually only weigh myself when I come (to the hospital).” “(The midwives) said I needed to start eating healthy. I just kept eating beef jerky, slushies. I didn’t eat bad food. I just ate what pregnant girls want to eat. I said, ‘If I’m getting bigger, that means my baby is growing. So, it doesn’t matter’.”	Daily weight monitoring and feedback on ideal weight trajectory FAQs on appropriate diet
Symptomatic bleeding or fluid loss	Confusion between spotting, miscarrying, and menstruation	“My pregnant sister is kind of nervous because she’s actually on her (menstrual) period now, and she doesn’t want to (have a miscarriage) again.”	Daily symptom assessment with feedback on need for immediate medical care (when appropriate)
Routine prenatal care	Barriers to transportation	“There were plenty of times (I missed appointments). I would normally have to catch a bus...and then I would have to walk up that big, long hill and then make a left...when (my belly) was getting out to here, I was like, ‘Ugh, I can’t do it anymore’.”	Complimentary transportation via Uber
Violence	Fear for personal safety during pregnancy	“(He) shot at me...because I used to date his cousin. He’s tried to come after me quite a few times, even after I gave birth.”	Diagnostic assessment of intimate partner violence and provision of assistance
Smoking	Perceived safety of smoking during pregnancy	“When you’re pregnant, it’s better not to stop (smoking) because the baby knows that you’re smoking and the baby can go through a nicotine withdrawal.”	Routine assessment of smoking and provision of smoking cessation resources
Preterm labor	Unfamiliarity with signs of labor	“My boyfriend, he had me laughing hysterically and I thought I was going into labor. I actually googled ‘laughing during pregnancy.'"	Contraction timer and feedback on preterm labor and delivery readiness
Fetal movement		“I always tell myself, ‘If I don’t feel her in the next hour, I’m going to the hospital’.”	Kick counter

**Table 2:** Pregnancy risk factors noted in literature, but not in these interviews.

Alcohol use	Drug use	Depression	Pregnancy history
Routine assessment of alcohol consumption and provision of smoking cessation resources	Routine assessment of drug use and provision of smoking cessation resource	Mood monitoring with triggered and routine assessment of depression	Baseline assessment of pregnancy history

### Risk Assessments and Communication

The MHP app gathered data regarding these risks factors through voluntary daily assessments. Deterministic algorithms then delivered patient-specific risk feedback and recommendations (eg, diet, lifestyle) tailored to individual users. For example, if the app detected a decrease in self-reported cigarette use, it provided encouraging messages in addition to quitting resources.

**Figure 1:**
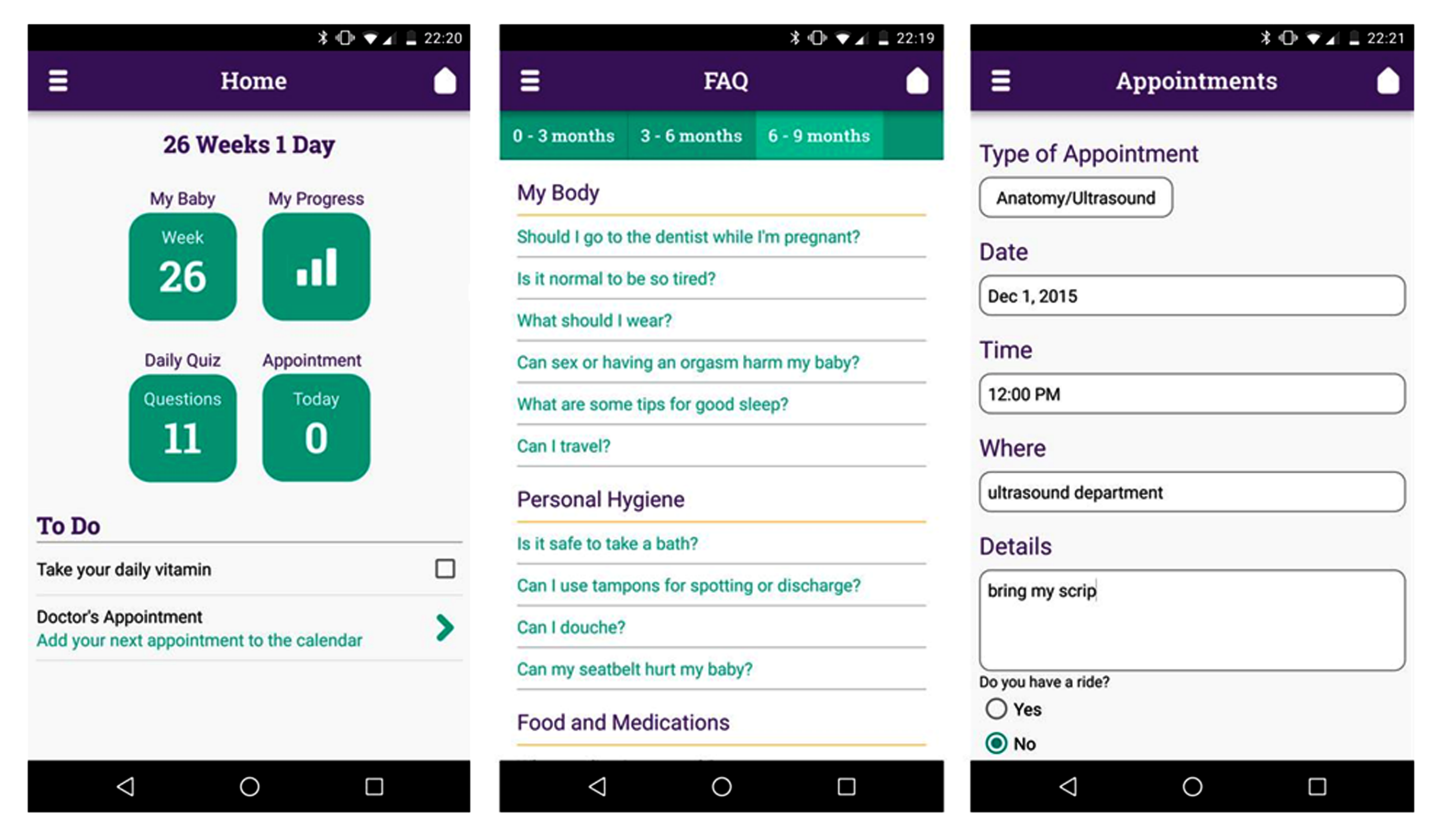
Sample screenshots taken from MHP, which was evaluated in proof-of-concept pilot with 16 women. From left: (1) Home screen, (2) frequently asked questions, and (3) appointment scheduling tool.

**Figure 2:**
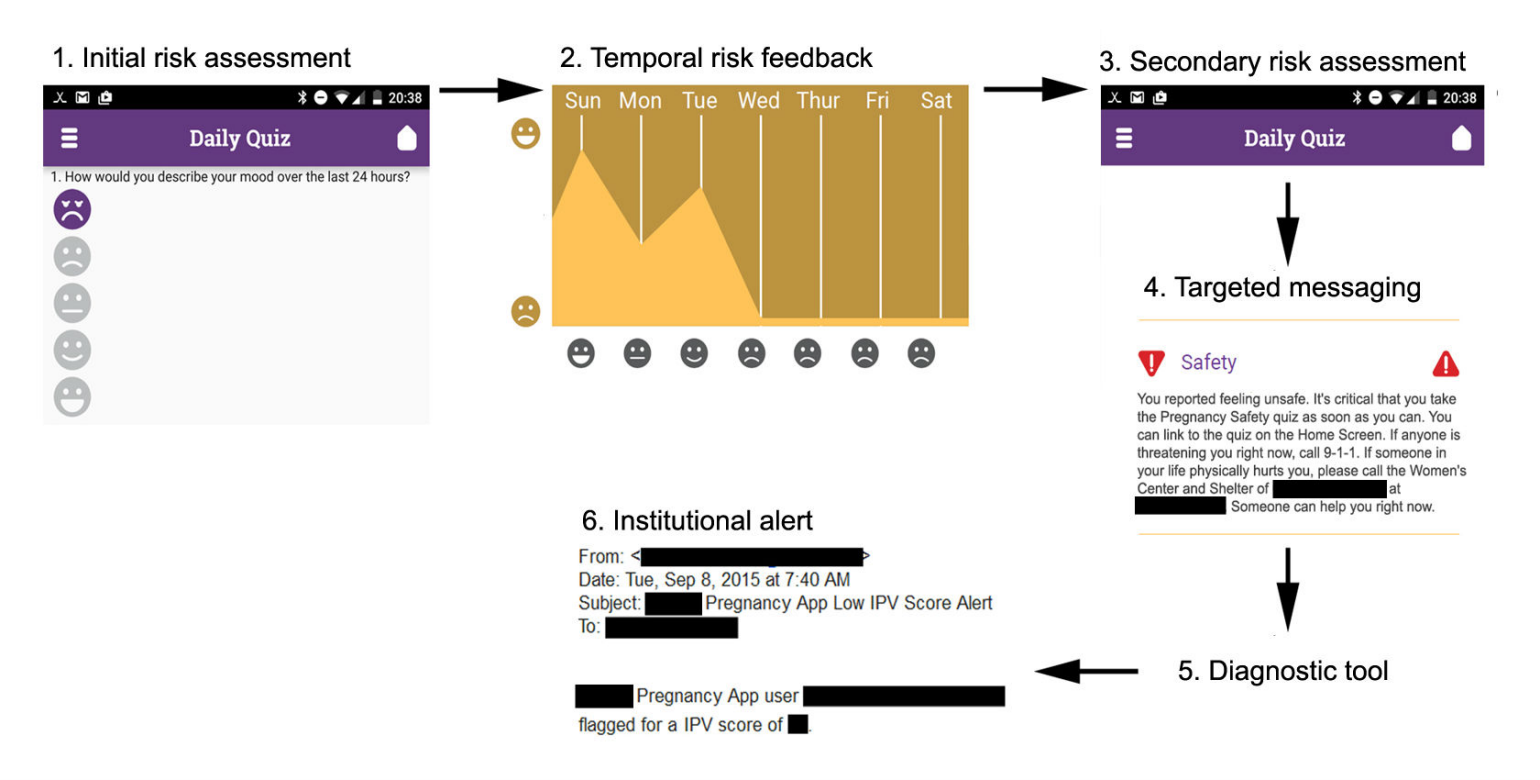
Logic diagram to identify, communicate, and intervene with a specific preterm birth risk (eg, intimate partner violence). From left: (1) user completes daily questionnaire, (2) user receives feedback on risk factor over time, (3) algorithm determines whether additional diagnostic questions are necessary, (4) user receives targeted messaging, (5) user completes validated diagnostic tool, and (6) physician receives real-time alert of intimate partner violence.

The app (Figure 1) also provided basic pregnancy education, reminders (eg, appointments), access to information and scheduling resources, and fetal health monitoring aids (fetal movement, “kick,” and contraction counters). To spur action, as soon as the app detected high-risk events, such as intimate partner violence, suicidal ideation, or clinical indicators (eg, preterm contractions), it sent real-time alerts to medical staff (Figure 2). Women were then contacted directly and linked quickly to appropriate medical or social services.

### Usability Evaluation

#### Pilot Study

After completing the design and pretesting of the MHP app, we conducted a 3-month proof-of-concept pilot (*N*=16) to assess its usability by women in all stages of pregnancy. As the app had features targeted to each pregnancy trimester, we aimed to enroll 5 users per pregnancy trimester. Potential participants were identified at routine prenatal appointments at the Magee-Womens Hospital outpatient clinic, which serves patients qualifying for Medicaid. Participants were recruited from August 31 to September 3, 2015. All participants remained in the study for 3 months or until they gave birth.

After providing informed consent and completing a baseline assessment, participants received a digital weight scale and a smartphone preloaded with the MHP app. All participants agreed to complete monthly phone interviews.

The proof-of-concept study was reviewed and approved by the Institutional Review Boards of both the University of Pittsburgh and Carnegie Mellon University. All participants provided written informed consent before participating. Interviewees received US $35 for their time. App pilot participants received US $15 for completing each of 3 telephone interviews over the course of the study and an additional US $25 if they completed each component (interviews and daily quizzes). In addition, participants kept the scale and smartphone.

### Measures

Participants completed daily quizzes on the app on a voluntary basis. A baseline quiz assessed baseline preterm birth risk and subsequent daily quizzes assessed the preterm birth risk factors identified in the normative and descriptive research (eg, mood, symptomatic bleeding or fluid loss, weight, and smoking, as detailed in Tables 1 and 2). Patients’ responses were used to provide them with risk feedback (eg, weight trajectory contrasted with ideal weight trajectory) and to gather data on factors that can predict preterm birth. Over time, the app used the patient-entered quiz data to prompt for additional diagnostic measures. When algorithms prompted additional diagnostics, such as an intimate partner violence assessment, these were also administered as part of the daily quiz. Engagement was measured by frequency of use of the app, including completion of the daily risk assessment quizzes and additional diagnostic quizzes.

Participants also completed the following measures at baseline in person and at 1, 2, and 3 months by mobile phone:

1. The Perceived Stress Scale [22]: a 10-item inventory measuring how stressful various aspects of their lives are.
2. Behavioral intentions: an inventory with 2 health behaviors (breastfeeding for 6 months postpartum and daily intake of prenatal vitamin during the pregnancy).
3. Usability questions: 2 items regarding the helpfulness of the app and any technical difficulties.

At the final interview, participants who had given birth were also asked to share any information they desired about their birth experience and their use of the app.

### Statistical Analyses

Our study was designed to evaluate the usability of the app, under normal conditions of a high-risk pregnancy, with no incentives for use (just for completing our instruments). As a result, our analyses focused on usage data, with suggestive indication of patterns. Given the low statistical power of these preliminary data, we used an alpha level of .1 for statistical significance, treating our analyses as exploratory. We used IBM SPSS Statistics 23.0 and R 3.3.1.

## Results

### Users

Table 3 summarizes participants’ demographics and baseline data. Of 17 participants consented, 16 subsequently enrolled and remained in the study. One participant consented but chose not to enroll due to a time conflict with the enrollment session. Analyses are conducted for all 16 participants.

**Table 3:** Proof-of-concept pilot self-reported demographics (N=16).

Variable		Median (range) or n (%)
Age (years), mean (SD)		24 (18-35)
**Race, n (%)**		
	African-American	11 (69)
	Asian Indian	1 (6)
	Hispanic/Latino	1 (6)
	Caucasian	1 (6)
	Mixed race, other	2 (12)
**Income (in US $)**		
	0-5 k	6 (37)
	5-9999 k	2 (12)
	15-19,999 k	2 (12)
	20-24,999 k	2 (12)
	25-29,999 k	1 (6)
	30-49,999 k	0
	50-69,999 k	0
	70,000 k or more	0
	Respondent did not know	3 (19)
**Education, n (%)**		
	Less than high school	2 (12)
	High school/GED	3 (19)
	Some college	9 (56)
	2-year college degree (associates)	2 (12)
	Bachelor’s degree	0
	Master’s degree	0
	Doctorate or professional degree	0
**Previous smartphone ownership, n (%)**		
	Yes	16 (100)
**Previously used a pregnancy app, n (%)**		
	Yes	13 (81)
	No	3 (19)
**Pregnancy planned? n (%)**		
	Yes	4 (25)
	No	12 (75)
**Gestational weeks at enrollment, mean (SD)**		24.5 (11-30)
**Had a previously very preterm birth? (<32 weeks), n (%)**		
	Yes	3 (19)
	No	13 (81)

Birth outcomes for all 16 participants can be found in Table 4.

**Table 4:** Birth outcomes for the 16 pilot participants.

Birth outcomes		Frequency
Ongoing Pregnancy		1/16
	Gave birth prior to study completion	0/1
Normal gestation (>37 weeks)		13/16
	Gave birth before study completion	7/13
Late preterm (34-37 weeks)		2/16
	Gave birth before study completion	2/2
Moderate (32-34 weeks) or very preterm (<32 weeks)		0/16
	Gave birth before study completion	0/0

### Engagement

On average, participants voluntarily logged into the app every 1.5 days to complete daily risk assessments, with the range of assessments completed being 16.7% to 100%. Once logged in, participants visited an average of 5 (SD 1.0) screens (eg, logging an appointment, viewing FAQs). At each monthly follow-up phone interview, participants were asked how helpful the app had been on a scale ranging from 1 (not at all helpful) to 10 (extremely helpful). The mean response at 1, 2, and 3 months was 9.0 (SD 1.15), 9.36 (SD 1.28) and, 9.25 (SD 1.0), respectively, with no difference in perceived helpfulness over time, *F*_1,26_=1.70, *P*=.20.

In order to capture usage patterns, we characterized each day in terms of whether the woman took a daily quiz on that day (which, as mentioned, occurred on two-thirds of the days). We predicted this dependent variable from baseline data and covariates available when the last quiz was taken, using a generalized mixed logit model with a compound symmetric variance-covariance structure for the errors (ie, varying intercepts per woman), allowing women’s responses to have a fixed correlation within person over time.

Table 5 reports the results of these separate, generalized mixed logit models. The strongest relationship was that women used the app more often at earlier weeks of gestation (Odds ratio, OR 1.07, 95% CI 1.02- 1.12, *P*=.005). The app was also used more by women when their daily mood (measured on a 5-point scale) was worse (OR 1.20, 95% CI 0.99-1.47, *P*=.08), when they were self-reported smokers (OR 4.00, 95% CI 0.93 -16.9, *P*=.06), and when they had a lower body mass index (OR 1.07, 95% CI 1.00-1.15, *P*=.05). App use was unrelated to whether women felt their baby move, had a greater weight, or were at a later stage of gestation at intake.

**Table 5:** Daily and baseline characteristics and their associated odds ratio of missing at least one day of daily quizzes given current levels of these characteristics using a generalized mixed logit model.

Characteristics	Daily/baseline	OR (95% CI)	*P* values	Sample size (quizzes)
Trimester at start	Baseline	0.60 (0.31-1.16)	.13	762
Weeks of pregnancy	Daily	1.07 (1.02-1.12)	.005	762
Daily mood	Daily	1.20 (0.99-1.47)	.07	759
Body mass index	Daily	1.07 (1.00-1.15)	.05	762
Obese (vs normal)	Daily	2.42 (0.98-6.03)	.06	762
Overweight (vs normal)	Daily	1.98 (0.53-7.47)	.31	762
Baby moved	Daily	0.81 (0.46-1.43)	.50	760
Current weight	Daily	1.01 (1.00-1.02)	.27	732
Smoking	Interval determined by baseline response	4.00 (0.93-16.9)	.06	762

### Appointment Attendance

Participants had an attendance rate of 84% (63/75) at prenatal appointments, including 3 non-routine appointments for risks surfacing during the pregnancy, compared with 50% for the non-participant clinic population. Attendance was even higher - 89% (31/35 appointments) - among those who scheduled Uber transportation to their appointments through the app. The total cost across patients for providing Uber transportation was US $537.35. The conservatively estimated direct cost of a missed routine appointment provided by the Division Director of Maternal-Fetal Medicine of the hospital system is US $300 per patient. Therefore, the provision of rides suggested an approximate cost savings of US $7,203 for 16 patients over 3 months (~US $450/patient).

### Communicating and Assessing Preterm-Birth Related Risk

No participants reported any of the following behavioral risk factors at baseline: intimate partner violence, depression, alcohol use, or illegal drug use. Two participants reported being cigarette smokers. Real-time data collection through the app over the course of 3 months identified 1 case of intimate partner violence, 2 cases of routine smoking, 6 cases of depression scores greater than 10 on the Edinburgh Postnatal Depression Scale, with one participant reporting suicidal thoughts, and 26 cases (2 participants) of illegal drug (marijuana) use. We pre-specified risk markers with potential clinical significance, such that the app electronically notified clinical members of our research team, thereby triggering appropriate clinical interventions. Table 6 tabulates the possible symptoms of preterm birth detected by the app. Algorithms also determined whether participants reporting certain symptoms were given messages that instructed them to call the clinic, go to the hospital, or engage in watchful waiting.

**Table 6:** Tally of preterm birth risk symptoms reported via the MHP app by trimester.

Symptoms reported	Trimester 1 (n events)	Trimester 2 (n events)	Trimester 3 (n events)	Percent of total events (N=693), n (%)
None	73	332	205	610 (88.0)
Cramping	0	4	32	36 (5.2)
Feeling contraction	1	2	32	35 (5.1)
Abdominal pain	0	2	6	8 (1.2)
Vaginal bleeding	0	1	1	2 (0.3)
Gush or fluid leak	0	0	2	2 (0.3)
Total (N events)	74	341	278	693

### Risk-Reduction and Health-Promoting Behavioral Intentions

#### Breastfeeding

Agreement with the statement “I will try to breastfeed my baby for the first 6 months” increased over the study period. The mean response on a 5-point Likert Scale of agreement at 1, 2, and 3 months, respectively, was 3.63 (SD 1.63), 4.00 (SD 1.30), and 4.06 (SD 1.48). Paired *t*-tests revealed significantly higher intentions to breastfeed compared with those at baseline (mean=3.50, SD 1.41), at both 2 months, *t*_13_=−4.16, *P*=.001 and 3 months, *t*_15_ =−2.76, *P*=.01.

#### Prenatal Vitamins

Agreement with the statement “How often do you think you will take a prenatal vitamin over the course of this pregnancy” stayed high over the course of the study among all users. The mean response, on a 7-point scale ranging from 1 (I definitely won’t take a prenatal) to 7 (I will take a prenatal vitamin every day without fail), was 6.31 (SD 0.87) at baseline, 5.81 (SD 1.56) at 2 months, and 5.57 (SD 1.99) at 3 months, respectively. These values were not statistically significantly different from one another.

#### Perceived Stress

Levels of perceived stress decreased from baseline and remained low over the course of the study. The mean response on a 40-point scale, with lower scores indicating less perceived stress, was 16.4 (SD 5.99), 13.1 (SD 5.89), and 15.0 (SD 7.71), at 1, 2, and 3 months, respectively, compared with the mean response of 16.81 (SD 6.06) at baseline.

#### Qualitative Feedback

Debriefing interview conversations with participants yielded three key insights. (1) They appreciated the risk feedback. (2) Many treated the app as a form of social support, with one participant stating, “(the app) was the only person in my life who asked me how I was doing every day.” (3) All reported wanting a similar app for the early stages of parenting, with one stating, “Please extend the information to after my baby is born (how much to eat, how long they should sleep for). Right now, I’m just asking friends and family...”

## Discussion

### Principal Findings

A persistent barrier to providing sound care during pregnancy is maintaining contact with women at highest risk. They often have difficulty making appointments. Once they do, health care providers may lack the time needed to learn about their conditions and convey the information most critical to their unique circumstances, especially when culture or socioeconomic differences complicate communication. As a result, health care professionals may fail to provide patients with information in a way that allows them to make informed decisions [23].

One solution to such imperfect patient contact may be virtual care, using mobile phone health apps. Targeting patients through an app poses unique challenges. The app must be engaging and both gather and provide accurate, medically relevant risk information. Although hundreds of pregnancy-related apps, performing a range of functions (from tracking symptoms to hospital bag checklists), exist, few have been developed through a scientific process. To the best of our knowledge, MHP is the first pregnancy app grounded in behavioral decision research that provides and gathers individualized preterm birth-related risk information.

We applied behavioral decision research theory and methods to create a smartphone app that could engage participants who typically face barriers to accessing routine prenatal care. Our app sought to take advantage of a delivery mode that circumvented the barriers faced by women at high risk by connecting them with the health care system in a way that served their needs for two-way communication. Results from our proof-of-concept trial show a high level of engagement among women recruited from a clinic serving Medicare recipients. Participants were compensated for completing the 4 study surveys, but had no financial incentive for using the app. Nonetheless, they logged on every 1.5 days on average over the course of the pilot study and consulted an average of 5 screens when they did. Moreover, they expressed uniformly high levels of satisfaction with the MHP app, with some describing it in terms such as being a “virtual companion.”

Although special caution is needed in considering individual differences with such a small, if intensively observed, sample, the app appeared to be used most consistently (Table 5) by women who might need it most, such as those early in their pregnancy, those with declining mood, and self-reported smokers. The candor and clinical value in their reports can be seen in cases that might otherwise be detected in a less timely manner. There were also individual incidents in which the app provided a vehicle for helping women who reported apparent cases of depression, intimate partner violence, and drug use. In these cases, and others, the app revealed risk information that was not captured at our study baseline or in routine clinical care.

We believe that our app represents an improvement over other pregnancy apps on the market as it reflects two features essential to effective applications. One is a patient-centered design, grounded in discussion with community leaders, formative interviews with individuals drawn from the target populations, and iterative pretesting in its development (in addition to the feedback from the proof-of-concept trial reported here). That sensitivity helped participants to share intimate information about their personal risk. The second feature is being grounded in behavioral decision research, whose theory and methods structured the four essential elements of the development process: normative analysis, with medical experts, providing authoritative, relevant content; descriptive research, with users, informed by the research literature; prescriptive interventions, informed by research on debiasing and risk communication; and evaluation, reported here. Those bodies of research provide a foundation for identifying and addressing misconceptions, such as the mistaken belief that vaginal bleeding is normal during pregnancy. We believe that these features allow us to exploit the technology so that, as our proof-of-concept pilot suggests, the MHP app can communicate and gather sensitive personal health risk information, including the detection of risks, at more frequent intervals than is possible by routine medical care.

We were also successful in increasing access to routine care. By providing Uber rides in real time, we could overcome the barrier posed by lack of transportation for low-income pregnant women who live in inaccessible neighborhoods or have difficulty planning. In addition, increased appointment attendance shows the potential of the MHP app for realizing significant savings to the health care system.

Our app is also designed to address the difficulty that health care providers often experience in communicating even simple recommendations for behavior change [7], especially to women at high risk. It uses language derived from conversations with women drawn from the user population, it acknowledges the practical barriers that they can face in implementing desired behaviors, and it repeats relevant information at frequent intervals. These features allow the app to provide both education and notification, two elements of successful behavioral change. Thus, the app’s daily reminders about prenatal vitamin use may have helped to sustain the high level of self-reported intentions observed throughout the study. Breastfeeding intentions also increased significantly over the study, across women in different stages of pregnancy, suggesting the value of repeating an audience-friendly message.

We note that our study was a proof-of-concept observational study, and not a randomized controlled trial (RCT), limiting our ability to infer causality. Next steps include conducting a RCT over the entire pregnancy and evaluating the effects of the MHP app use on behavioral and clinical outcomes, including adverse birth outcomes, in conjunction with other clinical measures of preterm birth precursors. We also note that our volunteer participants were likely drawn from individuals familiar with smartphone apps and looking for support during the pregnancy. Given the proliferation of mobile phone–based health communications, such familiarity should be increasingly common, creating greater opportunity to take advantage of their speed, convenience, low cost, and potential confidentiality. Our MHP pilot results suggest that smartphone apps are a promising step for providing personalized care to at-risk patients who are otherwise hard to reach.

## Abbreviations

MHP: MyHealthyPregnancy
IPV: intimate partner violence
RCT: randomized controlled trial
